# Territorial variance in the UK’s refugee politics and its consequences: Young Syrian refugees in England and Scotland

**DOI:** 10.1177/23996544231165440

**Published:** 2023-03-29

**Authors:** Gareth Mulvey, Dimitris Skleparis, Brian Boyle

**Affiliations:** 3526 University of Glasgow, Glasgow, UK; Newcastle University, Newcastle Upon Tyne, UK

**Keywords:** Social citizenship, devolution, integration, aspirations, youth, Syrian refugees, England, Scotland, UK

## Abstract

Access to social rights is crucial to refugee settlement and integration, and a whole range of social policy measures determine the limits on those rights. In the United Kingdom (UK) various relevant social policies are divided into devolved and reserved categories. This has resulted in a distinct territorial variance in social rights and welfare provisions within the country. The aim of this article is to explore how young Syrian refugees experience this territorial divergence in two jurisdictions: in Scotland, where they are part social citizens; and in England, where access to social rights is more limited. We use the prism of social citizenship as a means of examining the experiences of settlement and integration of Syrian refugees in the two nations. We draw out contrasts between these experiences and locate them within the interactions between the politics of welfare and refugee politics in the two nations. We argue that fine variances in England’s and Scotland’s social rights and welfare regimes have an impact on the settlement experiences of refugees. England’s less supportive regime fosters self-reliance and faster labour market integration among refugees. However, this environment pushes refugees in England to accept any job that they might find, no matter how precarious, or how far it may be from their educational qualifications, past professional experience or aspirations. This has overarching implications for refugees’ outlook on life and long-term planning.

## Introduction

Unlike many countries in continental Europe, the United Kingdom (UK) does not have ‘one’ homogenous immigrant integration policy framework per se (Scholten 2014). Instead, like in any multilevel state, responsibility for immigrant integration has been de facto decentralised to the country’s territorial governments, following the establishment of devolved legislatures in Scotland, Wales and Northern Ireland in 1998–1999. Devolution and migration in the UK intersect in several ways. While entrance to the UK is reserved to the Westminster Government the lives of migrants once here cross central and devolved jurisdictions, supporting Hammar’s (2006) contention of the difference between immigration policy (reserved) and immigrant policy (devolved). The UK government reserves power over immigration and asylum, including selection and admission and the power to determine the criteria for acquisition of nationality and citizenship. However, the Scottish government has acquired de facto power over immigrant policy since devolution is based on the retainer model, whereby any policy area that is not specifically reserved to the UK government is automatically devolved to the Scottish level.

Like in any multilevel and multinational state, the centre-periphery cleavage in the UK has generated the creation of competing nation-building projects, whereby the ‘centre’ seeks to institutionalise a dominant culture, while the ‘periphery’ tries to create or safeguard a minority identity for itself (Keating 2004). Differences in terms of the institutions of decision-making, the hard politics so to speak, are also joined with variations in soft politics. The language and rhetoric of the Scottish Government when compared to successive UK governments have been considerably more ‘migrant friendly’ and successive Scottish governments have supported increased migration to Scotland, largely for demographic reasons. This has been accompanied by some variations in terms of who is allowed access to specific services, and indeed the universality of these services. As we suggest throughout what follows, Scotland maintains some aspects of universalism in welfare provision while Westminster has adopted an increasingly stratified approach. However, as of yet, relatively little is known about whether differences in rhetoric and in the provision of services have any real impact in how migrants experience their lives in the UK.

This article analyses original survey data from interviews with young Syrian refugees in the UK. The aim is to explore whether and how territorial variance in welfare stratification impacts young Syrians’ lived experiences in Scotland and England. Thus, the focus is on access to social goods, and with it, experiences of social citizenship. Our data allows us to compare the experiences of young refugees (18–32 years old) who arrived after 2015 based on where in the UK they have been settled.

We argue that fine variances in England’s and Scotland’s social rights and welfare regimes have an impact on the experiences of refugees. England’s less supportive regime fosters self-reliance and faster labour market integration among refugees, which is best illustrated by the higher employment rates among young Syrians settled there. On the other hand, however, this environment pushes refugees in England to accept any job that they might find, no matter how precarious, or how far it may be from their educational qualifications, past professional experience or aspirations. Highly-qualified young Syrian refugees who have settled there, and those who entered the country via the asylum route seem to be significantly more negatively affected compared to their counterparts in Scotland. In contrast, Scotland’s more supportive environment towards refugees, particularly with regards to housing, and support in the form of English for Speakers of Other Languages (ESOL) provisions are shown to facilitate settlement (Karyotis et al., 2018). Indeed, young Syrians settled there consistently reported having benefitted more from several social welfare services and provisions compared to those in England. In turn, this environment enables resettled and asylum route young Syrians to seek their integration into the labour market at a different pace and helps highly-skilled refugees in Scotland to fulfil more seamlessly their career aspirations, albeit at a slower pace than those who are settled in England.

The article begins with some of the key theoretical debates about social citizenship, particularly questions of the divergence in welfare regimes between UK and Scottish Governments. Who has access to social rights and under what circumstances is a key issue around both welfare state construction and retraction. A stratified regime of rights exists for the UK population, and this regime sets the social parameters in which refugees must rebuild their lives and is thus a crucial part of the settlement process. Indeed, it could be argued that refugees integrate into an already highly stratified society, thereby essentially integrating into poverty and disadvantage (Bloch and Schuster 2005; Lindsay et al., 2010). We then provide a necessarily brief outline of the two primary means by which Syrians have entered the UK and come to be recognised as refugees; those who were resettled by the UK Government and those who went through the asylum system. We then compare the experiences of Syrian refugees in England with those in Scotland before pointing to some of the consequences and lessons of those variations, both for what they tell us about refugee settlement, and what they contribute to ongoing debates about devolution and social citizenship.

## Nation-building and social citizenship: Scotland and the UK

The discussion of what type of entities Scotland and the UK are is beyond the scope of this article, though debates about this are worth careful reading (see for example Keating 1997; Davidson 2000; McCrone 2001; Mitchell 2010). Of more importance here is the divergence between the governments of each, and we contend that there is evidence of nation-building in the Scottish context, and indeed arguably also in post-Brexit UK (Beland and Lecours 2008; Kymlicka 2011; Mycock 2012). The role of people in nation-building projects is critical. Hall (1992) suggests, for example, that people participate in the creation of the very idea of the nation while Frödin (2013) argues that this production process can create new solidarities, such as having and being seen to have the right to have rights. The contours of citizenship are subsequently subject to contestation and change as nations themselves and the rights they bestow alter. Some populations are deemed fully deserving, some partially deserving and some undeserving; citizens, denizens and helots for Cohen (1988). These categorisation processes are thus concerned with the states’ desire to define, and then redefine deservingness (Könönen 2018). Who are characterised as deserving is subject to change, as evidenced from over a decade of welfare retraction alongside the hostile environment. We also wish to add an additional layer of complexity to debates about rights by inserting the possibility of territorial variance in how this deservingness plays out and is experienced *within* a state, that of the UK. The continuum of deservingness of populations to social goods is best encapsulated by social citizenship.

Here we use Marshall’s (1950) conception of social citizenship, though we do not do so uncritically. Marshall identifies the different but linked components of citizenship, political, civil and social, all tied to the possession of rights. Political citizenship provides political rights in the form of suffrage. Civil rights concern questions of liberty such as freedom of speech and association. Social citizenship concerns social rights in the welfare state.

Marshall’s argument has been subject to attention and critique from its inception. Here we outline just a couple of criticisms of relevance. Marshall shows little interest *in* or concern *with* territorial variance, exhibiting both a normative and empirical support for a centralised British state, and his schema is thus unable to fully encompass the era of devolution, alongside welfare state retraction, one of the key changes in social rights and regimes in the UK over the past 20-years. There has been significant and justified criticism of the single male breadwinner model implicit in Marshall’s schema; an erasure of women from social citizenship, and the absence of minority communities are of importance. Of particular interest to this article is those who are absent, and, more specifically, the question of the social rights of non-citizens. Indeed, it could be added that the era of austerity has subjected many more to the violence of differential rights regimes that Marshall’s work does not engage with (Cooper and Whyte 2017).

For Taylor-Gooby (2008: 10) “social citizenship concerns the rights and duties associated with the provision of benefits and services designed to meet social needs and enhance capabilities, and also to guarantee the resources necessary to finance them”. Marshall argued that rights should provide equality of status, though not of outcomes, and it is this universality that supposedly produces equal social categories (Revi 2014: 455). In the context of austerity though, this universality has been under significant threat, particularly in England and the process has significant implications for migrant populations: “Migrants may fully participate in the economy of a nation without being able to enjoy its cultural and social benefits” (Revi 2014: 460). The existence of non-citizens presents a challenge to Marshall’s citizenship, as it complicates the mythology of a single equality of status (Ibid). Instead, non-citizens become part of an increasingly racialised and classed system of citizenship rights (Turner 2016). That is, the notion that citizens have the right to the full gamut of social and political rights has been challenged by a long process of rights retraction and an accompanying rhetoric reminiscent of Victorian notions of deserving and undeserving poor.

Nevertheless, the gap between the rights of denizens and the rights of citizens of a specific state is real and can have territorial variance (Hepburn and Zapata-Berrero 2014). In the British context, devolution provides the institutional form to this variance. For Keating (2009: 97) “while true Scottish or Welsh social citizenship may or may not evolve, ‘the old British model’, captured by Marshall at the moment of its completion, is becoming a thing of the past”. So, devolution for the first time offers the opportunity for citizenship to be territorially differentiated (ibid: 98), with the various parts of the UK conceiving of it according to their own forms of ‘domestic’ policy, or indeed nation-building projects. Sanghera et al. (2018) remind us that practices of citizenship are contingent and situated within the everyday, as part of an ongoing process of negotiation. They suggest that focus be directed more on what rights do as social and political practices rather than what they are (Sanghera et al., 2018). To do this, understanding the lives of those experiencing these social and political practices is crucial.

The focus of this article then is on a population most subject to denizenship: refugees. In looking at welfare regimes and the changing contours of rights for this population in a UK context, we see divergences and a malleable rights settlement. But in all cases, we also see refugees denied full political citizenship rights, though the Scottish Government recently extended voting rights to refugees in Scottish and Council elections. Nevertheless, it is in the social rights of refugees that differences between Westminster and Holyrood are most evident (Mulvey 2018). Thus, a major aim of this article is to compare refugee experiences in two jurisdictions, one where refugees are part social citizens and one where their social citizenship is more limited. The key research question is, *how have different governmental approaches to social citizenship been experienced by young Syrian refugees*? Or alternatively, what are the differences in how young Syrian refugees experience their social rights in England and Scotland? We seek to contribute both an understanding of how denizenship is experienced, how it varies across space, and how these experiences help us to understand social citizenship in a devolved context.

The relationship between social policy, social citizenship and devolution is one that has been subject to considerable argument and debate. Indeed, the possibility for a generous form of social citizenship for some is predicated on a strong central state, with devolution seen as threatening the ability to redistribute social resources. This argument has been most famously made by Bogdanor (1999), who argued that devolving or federalising a state would ultimately undermine the welfare state. The argument revolved around the idea that local particularities can be flattened only by a unitary and/or central state, where need rather than location is the only driver of policy and practice. Keating argues to the contrary: that there is no automaticity about the relationship between welfare solidarity and devolution. For him “a less prejudicial way of proceeding would be to ask just what forms of citizenship and nationality are developing at the state, sub-state and trans-state levels and how these might be consistent with our normative ideas of solidarity and social citizenship” (Keating 2013: 158). Our contention is that the UK and Scottish Governments conceive of social citizenship differently, and this in turn has led to a different framing of immigrant policy. “‘Who’ enjoys the status of citizen, ‘what’ exactly that equality means and ‘which’ governments are responsible for delivering the services. The answers to those questions radically change the nature and meaning of social citizenship by questioning both its reality and its future” (Greer 2009a: 198).

Scotland has some different principles for welfare provision to both citizens and denizens, which has resulted in greater access to social rights for those who live in Scotland compared to England (Hepburn 2016). In the area of health, for example, care for the elderly, free prescriptions and eye-care are available to residents in Scotland, in contrast to those who live in England (Greer 2009b; Keating 2009). The primary divergences have been around selectivism versus universalism, where changes have mostly been England changing and Scotland (and Wales) not. Thus, as Keating (2005) points out, these processes do not reflect a radical departure on the part of the Scottish Government, but that instead it is clinging to the liberal values of the social democratic welfare state.

There are divergences also in the way social security is both conceived and implemented. Scottish policy-makers remain wed to elements of the welfare consensus, which saw poverty as being largely about lack of money. It therefore has used some of its extra powers emanating from the 2016 Scotland Act to mitigate the bedroom tax and cuts to Universal Credit. The relationship between the national and the local also differs with the Localism Act (2011) attempting to sever the link between the Westminster Government and any responsibility for social housing, unlike in Scotland. These have broader impacts but are also exemplars of the divergence mentioned above.

Beland and Lecours (2008) agree that divergence is primarily about a succession of Westminster Governments breaking with the post-1945 welfare consensus, with conditionality of rights to social goods replacing universalism. For Wincott (2009) the attack on the values inherent in the welfare state since Thatcherism served to both undermine social citizenship and create devolutionary pressures. Thus, the centralised UK state, as it retracted from social citizenship and became less able to flatten out inequalities, was challenged at the sub-state level.

The point of interest here is to highlight what divergences in social citizenship and welfare mean for those without full citizenship. In the case of Syrian refugees in the UK, access to social rights links to broader debates about social citizenship and the welfare state after devolution. It is our contention that the focus on a British model of social citizenship, and indeed a national model of migrant settlement, belies contestation about what the national is and downplays variation *within* the state. That is, who has access to social goods in the form of social policy, the very epitome of social citizenship, can have territorial variance within national states (Koopmans and Statham 1999; Hepburn and Zapata-Berrero 2014).

This clearly raises fundamental questions about how rights negotiations are inclusive or exclusive of migrant populations. The relationship between social citizenship, devolution and immigration is, however, an extremely complex one, with immigration being both transversal and multi-level, so both vertical and horizontal. Access to political citizenship is reserved to Westminster, whereas access to many aspects of social citizenship is devolved. Citizenship, nationality and immigration control are reserved to Westminster, but the Scottish government retains control over health, housing, education, economic development, policing and culture. And overall Syrian resettlement and the asylum process are reserved, but the Scottish government and Scottish Local Authorities have their own policies and practices around settlement.

As devolved authorities gained powers, and as Westminster moved away from the welfare state consensus, space opened up for difference in devolved administrations particularly around the move towards more conditionality in social rights in England, governed by Westminster. For instance, as part of the ‘New Scots’ Refugee Integration Strategy, the Scottish government has proposed that asylum seekers be granted full civil rights, including the right to employment, in a similar fashion to refugees. In contrast, the Westminster approach excludes asylum seekers from labour market access until they have officially been granted refugee status.

In the area of education, there is no UK-wide strategy for asylum seekers and refugees beyond the age of compulsory schooling (i.e. 16 years of age). The main contrast concerns tuition fees for further and higher education, which home students in Scotland do not pay. The Scottish Funding Council (SFC) waives the fees for asylum seekers and refugees who attend college, and study a full- or part-time ESOL course or other part-time advanced or non-advanced course (Scottish Government 2013: 57).

Könönen (2018: 56) also points to the direct links between immigration and experiences of welfare, as “immigration controls and welfare controls can be conceived of as separate institutions, yet they are intertwined, as the type of legal status directly affects a person’s eligibility for welfare services” (see also Bloch 2008). The stratification of welfare rights for those without – and, indeed, for many with – full citizenship rights is indicative of these links. The stratification of rights at the Westminster level, for migrants and in many cases also non-migrants (see Wright et al., 2020 on welfare conditionality; see Turner 2016 on denizenship) is a process of derecognising those people as political actors. How this plays out in terms of lived experiences of Syrian refugees in England and Scotland is what we now turn to.

### Syrian settlement in the UK: resettlement and asylum routes

While it is not possible to put a simple start date for what unfortunately came to be described as the ‘refugee crisis’, from spring 2015 the movement of Syrians into and across Europe increased and led to a variety of state responses. The UK, sitting on the geographic periphery of Europe, and with the ‘benefit’ of being an island situated somewhat apart from the debates raging on the mainland followed “a distant or non-proximate approach by increasing securitization at the borders and outsourcing border controls and aid to other countries” (Ibrahim and Howarth 2018: 353). This likely would have remained the strategy had it not been for public and political pressure.

Solidarity movements emerged organically across the country (Piacentini 2016), most visibly after photos of Alan Kurdi donned the pages of even the most xenophobic newspapers. This sat alongside parliamentary politics, seen for example in the government’s unwillingness to openly oppose the Dubs amendment, an amendment that would allow much easier access to the UK for children through resettlement. Such pressures wrongfooted a government convinced that years of vilifying ‘bad’ migrants made immigration a safe policy area for them, and so in autumn 2015, Syrian refugees began to arrive in the UK as part of what became known as the Syrian Vulnerable Person’s Resettlement Scheme (VPRS). The Government set a target of 20,000 arrivals over a 5-year period.

Syrian nationals resettled under the VPRS are identified by the UNHCR in refugee settings in the MENA region (Middle East and North Africa) according to eligibility criteria agreed with the UK Government. The UK Government took a specific decision to only resettle the ‘most vulnerable’ Syrian refugees in camps adjacent to Syria, meaning those arriving in the UK via that route were specifically ‘selected’. Following initial assessment, refugees are referred to the UK Government for further screening and to the International Organisation for Migration for a health assessment. Pending clearance, the Home Office then refers them to local authorities across the UK who have indicated a willingness and capacity to participate. These local authorities indicate who they are willing to resettle and are then responsible for delivering the programme in line with the Home Office Statement of Requirements (SoR). Local authorities receive funding for everyone they agree to resettle, and they may choose to deliver core services in-house, or commission other providers. Additional funding can be requested from the Home Office where there are complex health or social care needs. In theory, therefore, while implementation will and does vary across local authorities, they should all have some common factors in how they go about resettlement due to the SoR.

The Scottish Government and Scottish local authorities were among the first to commit to resettling refugees in this way. The First Minister held a Refugee Summit in Edinburgh and convened a Refugee Taskforce in early September 2015. 31 of 32 Scottish local authorities agreed to take part. By the end of March 2020, 19,768 refugees had been resettled under the VPRS, which was paused soon after (Refugee Council 2020); 17% of the total settled in Scotland, despite Scotland having just 8% of the UK population (STV 2019).

In addition to resettled refugees, there are also those who were either in the UK on the outbreak of the Syrian war and subsequently applied for refugee status, and those who fled Syria and managed to find their own way to the UK where they will have had to go through the asylum process. Between 2011 and 2017 around 8000 Syrian asylum seekers and their dependants had been accepted as refugees after going through the asylum process (Full Fact 2018). They receive a very different welcome, having to first traverse the often long and convoluted asylum process, during which time they can be sent to any part of the country as decided by the Home Office’s dispersal policy (Karyotis et al. 2021).

In theory, therefore, it is among Syrian refugees who came through the asylum process that most differences in policy and practice should be evident. Successive British Governments have been clear that they do not support the integration of asylum seekers, at least in part due to operating within a ‘culture of disbelief’, believing most asylum seekers to be economic migrants wishing to subvert ‘normal’ immigration controls (Yeo 2020). This has meant in practice that access to social goods for those in the asylum process has been subject to successive waves of restrictions (Darling 2016; Squire 2009).

Scottish Governments have made a lot of their more ‘progressive’ stance on refugee issues than that of Westminster, and this position has been a cross-party one, with the SNP Government following some similar positions taken by the previous Labour/Liberal Democrat coalition administrations. What Scottish administrations have argued is that where they have the powers to do so integration should begin on the day of arrival (Scottish Government 2013: 1), and so more generous social provision has been offered to those in the asylum system in Scotland than those in England. For example, those in the asylum process in Scotland can access further education, though it is restricted to an arbitrary figure of just 14 hours per week. Those recognised as refugees in Scotland are not required to show a ‘local connection’ to access social housing, unlike in England.

## Young Syrian refugees in Scotland and England

Our empirical analysis explores how territorial divergences in social citizenship and welfare between Scotland and England affect the experiences of young Syrian refugees who have settled there through both resettlement and asylum system routes. Our findings are based on 477 surveys conducted with Syrians between the age of 18 and 32 from July to September 2017.^1^ Six young Syrians living in England and seven in Scotland, all native Arabic speakers, were recruited and trained as peer researchers, privileging female (10) over male (3) researchers, to minimise known barriers to survey participation by female migrants (see Hamilton 2015). Two of our peer researchers were based in Coventry, one in London, one in Manchester, one in Sheffield and one in Newcastle. Of those in Scotland, one was based in Aberdeen, one in Edinburgh, and five in Glasgow. This design was guided by the fact that Scotland had taken in more than a third of all UK’s Syrian refugees (see Addley and Pidd 2016), and the UK government’s policy of ‘dispersed accommodation’, which has resulted in specific local authorities hosting disproportionately large numbers of refugees and asylum seekers to their population (see Lyons 2017).

Due to the lack of publicly available official data regarding the demographic characteristics of the Syrian population in the UK, convenience sampling was employed. The surveys were conducted mainly through Computer-Assisted Personal Interviewing (CAPI), which accounted for 77% of completed interviews. Tablet devices loaded with an offline version of the Qualtrics survey software were used to conduct the face-to-face interviews. Computer-Assisted Self Interviewing (CASI), which amounted to the remaining 23% of our sample, was also used to further maximise our geographical reach and number of respondents. A link to an online version of the questionnaire was generated and shared with local authorities and the personal networks of fieldworkers.

When combined, the overall dataset produces a fairly balanced split in terms of our respondents’ nation of settlement, with 44% (212) living in England and 56% (265) living in Scotland at the time of the survey. There is a slight imbalance based on mode of access to the UK, with proportionately more of our Scottish sample having come through resettlement (55% in Scotland; 39% in England). In part, this is explained by the greater degree of engagement with our project among local authorities in Scotland. In both nations, males comprised the majority of our sample (62% in England; 66% in Scotland).

The following section explores the relationships between these variables and makes use of chi-squared tests and log-linear analysis to test the statistical significance of any apparent associations. The relationships discussed below were found to be statistically significant at the *p* < .05 level unless explicitly stated otherwise.

## Access to social rights: social welfare support

There has always been a difficult and often politically motivated balance to be found between providing better provision for refugees in relation to help and support and encouraging resilience. Research to date has indicated that refugees, and indeed many other migrants often make strategic calculations about immediate labour market access, or pursuing training and education with the aim of a better and often more appropriate job (Jayaweera and Anderson 2008; Mulvey 2013). Successive UK Governments have adopted an approach that immediate work, any work, is preferable to training or education. This process is a ubiquitous one on the part of the UK Government, visible in many of their employability programmes whereby the quality or security of jobs are secondary to getting people into any kind of work. The employability approach places responsibility on the individual to obtain and keep a job. Nevertheless, debates about a continuum between self-reliance and institutional support are complex and hugely political. These link to issues of social citizenship practices, access to rights and the right to have rights as well as welfare state retrenchment. Social citizenship practices in Westminster and Holyrood have exhibited some contrasts. What has not yet been known is how this plays out at the level of individuals and communities.

What we see from our participants in terms of reliance is that young Syrians, regardless of the route of access, rely more on the state and municipal authorities for housing in Scotland than in England (69% against 43%). Only a small minority (17%) relies on its own resources to cover accommodation expenses in Scotland, while in England young Syrians appear to be more financially self-reliant (35%). This might be directly related to the fact that residents in Scotland enjoy, in principle, better rights to housing than those in England, after new homelessness legislation was passed by the Scottish government in 2012. In addition, what constitutes reliance here is an open question. However, we suggest that the greater preponderance of the overall Scottish population living in social housing is part of the story, suggesting that the Syrian population in Scotland are reflective *of* rather than contrary *to* that broader population’s housing status. Another key aspect concerns whether people are sharing accommodation or not. Of those in England, 30% report that they live in shared accommodation, while for those in Scotland, this figure is just 11.5%. This is linked to the previous point: greater support available in Scotland allows for decision-making that can include independent living like the rest of the Scottish population, and the greater availability of social housing ensures that this is a more realistic proposition.

These cross-national differences also persist when it comes to other forms of support. Government welfare payments were the main source of income for young refugees in Scotland (65%), while just 35% of those in England reported this as their primary source of income. This plays directly into debates on reliance versus support, with the nascent Scottish state apparently more willing to provide various forms of support to refugees. Young Syrians in Scotland also consistently reported that they have benefitted more from a number of welfare provisions compared to those in England, most notably from English language courses (74% against 54% respectively).

It is also worth mentioning that the differences in terms of social welfare support between those who arrived via the asylum route and those who were resettled were unsurprisingly stark. However, the relationship between mode of access and social welfare did not differ significantly between the two nations. In both home nations, asylum route refugees were less likely to have benefitted from state-subsidised accommodation, non-shared accommodation, government welfare payments, and ESOL courses compared to their resettled counterparts – a clear indication of a UK-wide two-tier system of refugee support.

## Access to social rights: work

As mentioned earlier, Holyrood and Westminster have contrasting approaches to when refugees should be recipients of social rights. This manifests two opposing rationales towards integration: for Holyrood, it should begin with the asylum seeker’s arrival in Scotland; for Westminster, it should begin when and if an asylum seeker becomes a refugee. Scotland’s rationale stems from a bigger argument that sees immigration as necessary to maintain demographic and economic growth amid an ageing population and increasing key gaps in the labour market (Scottish Government 2020). The same issue in England has developed an increasingly divisive anti-immigrant tone (Wallace 2018). For the time being, asylum seekers in both home nations do not have the right to employment. They become eligible to work on the same basis as British citizens only once a final positive decision on their application has been made.

Refugees in the UK experience significantly lower employment rates compared with minority ethnic groups more broadly (Bloch 2004), and way lower than the average national employment figure, which at the time of our survey stood at 75.1%. Our findings show that Syrian refugees in Scotland are further away from labour market integration than those in England (see Table 1). Perhaps this also helps explain why the former tend to rely much more on government support/welfare as their main source of income than the latter, as mentioned earlier. More specifically, 34% of our respondents in England reported that they were in some form of employment, compared to just 20% of those in Scotland. More than one in five young Syrians in Scotland said that they were unemployed and looking for a job (22%; 15% in England). Despite their better labour market participation rates, young Syrians in England appear to face significantly higher precariousness in their employment than those in Scotland. Indeed, 53% of those in employment in England reported that they did not have social security, compared to 30% of those in Scotland. Employment and an increasingly conditional welfare system tie work to social citizenship in an explicit way. The curtailment of social welfare provisions in England pushes young Syrian refugees to enter the labour market faster than those settled in Scotland. This forced self-reliance, however, exposes the former to worryingly precarious employment conditions.

**Table 1.** Employment status by nation of settlement, mode of entry, and gender.

Nation of settlement (N = 472)	England, %		Scotland, %	
Employed	34		20	
Student	32		36	
Homemaker	10		17	
Unemployed, looking for a job	15		22	
Unemployed, not looking for a job	9		5	
		
Nation of settlement and mode of entry (N = 383)	Asylum route, %	Resettlement route, %	Asylum route, %	Resettlement route, %
Employed	48	19	36	4
Student	24	42	37	37
Homemaker	4	15	9	23
Unemployed, looking for a job	18	18	16	31
Unemployed, not looking for a job	6	7	3	4
		
Nation of settlement and gender (N = 467)	Male, %	Female, %	Male, %	Female, %
Employed	41	24	26	9
Student	31	37	39	30
Homemaker	0	28	0	49
Unemployed, looking for a job	19	8	31	6
Unemployed, not looking for a job	9	4	4	6

When it comes to the types of jobs young Syrian refugees are employed in, almost an equal share of those in England and Scotland reported that they were employed in professional/highly technical jobs (23% and 25% respectively). While there was a small gap between the two countries in terms of the proportion of young refugees employed in skilled manual work (28% in Scotland and 17% in England), this difference was not statistically significant. As we explain in the following section, these employment patterns partly reflect and partly contradict the different qualifications that Syrians in each of the two home nations have.

Alongside these patterns, when it comes to employment there were also notable differences in terms of both gender and route of access into the country. Although the relationship between gender and employment does not seem to depend on the nation of settlement, a clear gender divide was present, with male refugees being significantly more likely to report they were in some form of employment (32%), in comparison to females (16%). This is in line with previous research which suggests that childcare responsibilities tend to impact disproportionately female refugees’ economic activity (Bloch 2008).

The starkest difference, though, arises from the relationship between an individual’s route to refugee status and employment rates. Young Syrians who arrived via the asylum path were significantly more likely to be employed (42%), compared to those who were resettled (9%). This relationship was also significantly stronger in Scotland, where the odds of being employed at the time of the survey were 13 times higher if refugees came from the asylum route versus resettlement, while the odds of being employed for those via the asylum versus resettlement group was just 3.8 times greater in England. From this we can infer that Scotland’s slightly more supportive social welfare environment enables resettled and asylum route refugees to seek their integration into the labour market at a different pace. This is not so the case for those in England, where the relative lack of social support presents the two groups with an almost equal urgency to access work quickly and become independent.

## Access to social rights: education

**Figure 1.**
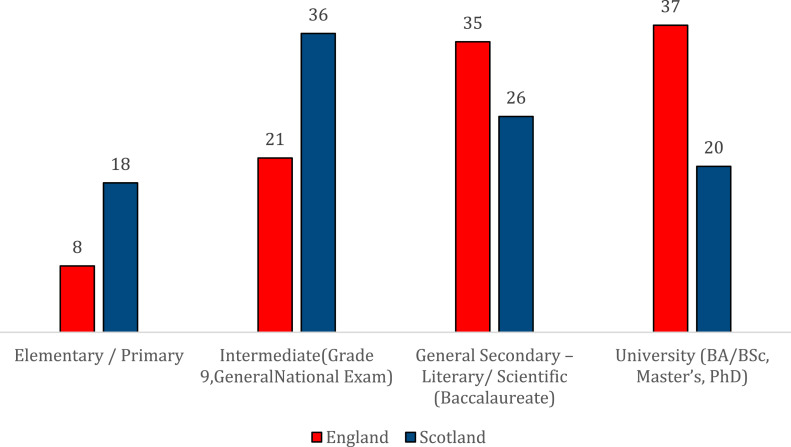
Highest achieved educational qualifications of young Syrians in England and Scotland (%).

Apart from the UK-wide two-tier system of international protection and support (Karyotis et al. 2021) and the policy of forced self-reliance in England, the differences in the educational backgrounds of young Syrians in England and Scotland explain much of the divergence in employment rates. Those in England are more likely to have documentation that proves their educational qualifications (63% in England; 40% in Scotland). Evidence of educational qualifications is essential for much employment and helps to explain why those in England demonstrate higher employment rates than their counterparts in Scotland. Moreover, 37% of those in England were university educated, in comparison to 20% of young refugees in Scotland (Figure 1). Based on this, one would expect that the share of young Syrians employed in professional/highly technical jobs in England would have been higher than that of their counterparts in Scotland. Yet, as we demonstrated above, this was not the case. So, while young Syrian refugees in England are more likely to be in employment, those with a Higher Education degree, i.e., those more highly-skilled, are also more likely to be underemployed relative to their educational qualifications, in comparison to those settled in Scotland.

When it comes to pursuing education once in the UK, there were no statistically significant differences in relation to the nation of settlement, route of access, or gender. A slightly higher proportion of respondents in Scotland reported that they were students at the time of the survey (36%) compared to those in England (32%), while 39% of those who entered via resettlement were students, compared to 31% of those from the asylum route. Both male and female young refugees displayed similar rates of student enrolment (36% men, 33% women). The absence of a statistically significant relationship between enrolment in education and nation of settlement might come as a surprise given Scotland’s slightly more inclusive education environment. Yet, one must acknowledge that other key factors may also come into play here, such as a refugee’s age, level of English language skills, proof of previous educational qualifications, length of stay in the UK, as well as their past education experiences and future aspirations. It is the latter that our analysis now turns to.

## Aspirations, past experiences and present realities

In this final part of our empirical analysis, we explore the career and education aspirations and past experiences of young Syrians in England and Scotland, and the extent to which these are generally in line with the employment, job and education patterns identified above. Previous research has identified a clear mismatch between pre-migration and current employment rates of refugees in the UK, which tends to disproportionately affect the highly-skilled and those who traversed the asylum system (Bloch 2008). Despite some similarities between our two populations – in employment and education aspirations and the extent to which the latter correspond to past and present experiences – we observe some important territorial divergences. We argue that these clearly emanate from territorial variances in social citizenship, which, in turn, tend to impact more negatively highly-skilled young Syrian refugees in England, and particularly those who entered the country through the asylum route.

In terms of integration into England’s and Scotland’s education systems, young Syrians’ student status appears to be more or less in line with both their past and aspired status. There was very little variation in both past experiences and educational aspirations between the populations in the two nations. Around one-third of respondents indicated they aspired to student status, while just under 40% reported they were in education in the 6-months prior to leaving Syria. This also aligns with the pattern of current student status discussed in the previous section, where there were no significant differences between those settled in either nation.

**Figure 2.**
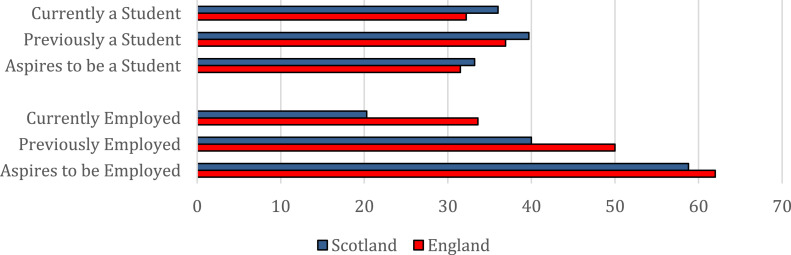
Present, past and aspired student and employment status among young Syrian refugees in England and Scotland (%).

Moving on to employment, it appears that refugees’ current employment status falls far short of both their past experiences, and future aspirations. While this is true across both nations, there seems to be an even greater mismatch when it comes to those settled in Scotland compared to those in England. More specifically, a majority of young Syrians in both England and Scotland aspired to some form of employment (62% and 59% respectively - Figure 2). Despite these similar aspirations, however, there were some clear areas of divergence between the two nations. Young refugees in England were significantly more likely to both have been in employment in the 6-months prior to leaving Syria, and being currently employed (as discussed previously), compared to those in Scotland. As well as looking at the differences in overall rates, we can also compare the relative mismatch between young refugees’ past experiences, future aspirations, and present employment prospects. With regards to past versus current employment, of those who were in some form of work in the 6-months before leaving Syria, only 41% were currently employed in the UK. There were also clear differences at the national level here, with individuals who were previously in employment and settled in Scotland being less likely to be currently employed (34%) than those also previously in work, but who settled in England (48%). Moving on to young refugees’ aspirations versus current employment, of those who aspired to be in any type of employment in the future, just 39% were currently in work. Again, those in England were more likely to have their current employment status match their aspirations, with 54% of those who aspired to be in employment currently working, compared to just 27% of those aspiring to full-time employment in Scotland.

**Figure 3.**
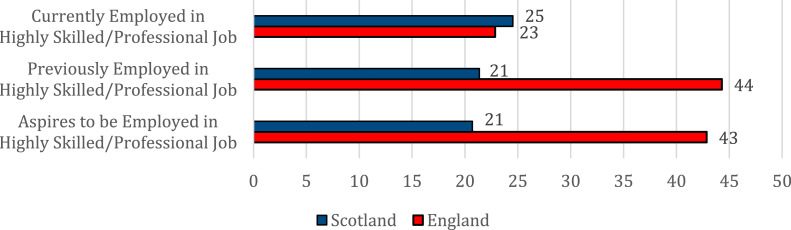
Present, past and aspired highly skilled/professional job status among young Syrian refugees in England and Scotland (%).

To be sure, both cohorts’ full-time employment status at the time of the survey appears to be very far from both their past, as well as their aspired full-time employment status, particularly for those in Scotland. However, when we examine these patterns further, and include specific types of jobs in our analysis, this picture changes slightly. Young refugees in England were significantly more likely than those in Scotland to both have previous experience in highly skilled or professional roles (44% vs 21% in Scotland), as well as aspire to work in this type of jobs in the future (43% vs 21% in Scotland), as shown in Figure 3.

Yet, despite this variation, there was no significant difference in the percentage of young Syrians currently employed in highly-skilled jobs between the two nations, as discussed previously. Thus, highly-skilled young refugees in England are more likely to be underemployed relative to their past job experiences and aspirations, compared to those in Scotland. Simply put, the ‘better’ employment participation figures in England must be placed within the context of highly-qualified Syrian refugees there doing jobs below those that they have been trained to do (as we demonstrated earlier), they have done in the past or can aspire to in the future.

**Figure 4.**
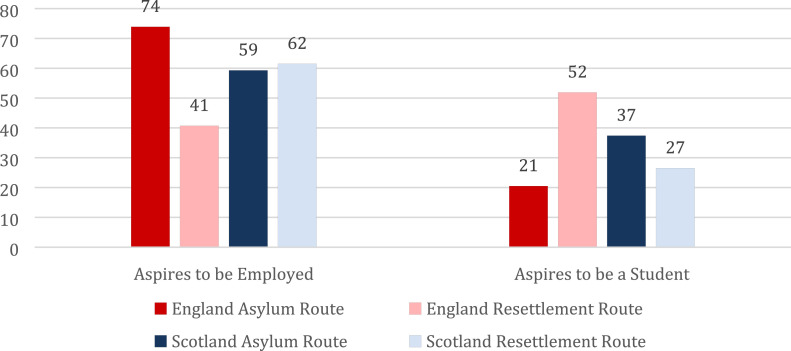
Aspired employment and student status among young Syrian refugees in England and Scotland by mode of access (%).

Finally, bringing the dimension of an individual’s pathway to refugee status into the discussion, reveals additional important differences that further substantiate our previous arguments. What is worth mentioning, is the significantly higher share of asylum route refugees in England who aspire to any type of employment compared to those who have been resettled there (74% against 41%) (Figure 4).

In contrast, state-provided support enables those resettled in England to aspire to student status at much higher rates than those who came through the asylum route (52% against 21%). Again, these findings could be suggestive of the urgency with which asylum route refugees are faced to make ends meet within the context of limited state support in England. Meanwhile, for those in Scotland, there were no significant differences in either employment or student status aspirations across the two settlement routes, testament, perhaps, to the Scottish government’s desire to treat refugees similarly irrespective of their mode of access to the country. In fact, asylum route refugees in Scotland reported a slightly higher desire to pursue a degree than those who have been resettled there (37% against 27%), however the difference was not statistically significant.

## Conclusion

Legally resident migrant populations do not experience social citizenship equally (Corrigan 2010). Simply having rights is therefore not adequate (Anderson and Hughes 2015). Migrants are users and not abusers of welfare, and so the restrictionist practices currently in vogue across many governments are based on a false premise, and, what is more, have long-term negative impacts in terms of socio-economic inequalities (Corrigan 2010: 433).

In this article, we attempted to examine some of those impacts. What we see is two diverging welfare regimes, one that treats refugees, and especially those who came through the asylum system, as denizens at best, and one that provides a little more in the way of social rights. This allows refugees in each respective nation to aim for self-reliance at different paces. In England, the relative lack of social support places the onus on refugees to access work quickly and become independent. This generates some important consequences for refugee populations settled there, which are not, nevertheless, uniform. Although young Syrians in both England and Scotland are significantly underemployed compared to what they were used to back in Syria and what they would have hoped for here in the UK, it is particularly those in England who tend to be employed in precarious jobs, and, at times, far below their qualifications, past experiences, and aspirations. Highly-skilled young refugees and those who entered the country via the asylum route seem to be disproportionately negatively affected compared to their counterparts in Scotland.

The urgency to make ends meet in England, in contrast to the forms of limited social support and space in Scotland, leads to different experiences of settlement, and thus, impacts on experiences of social citizenship. These, in turn, feed into how refugees evaluate the role of key institutions/actors and people that they have encountered in the UK. And they also shape young refugees’ outlook on life in the country.

More specifically, 89% of refugees in Scotland evaluated positively the national government compared to 70% of those in England, although clearly what constitutes the ‘national’ in the Scottish case is not necessarily clear. Looking at another tier of government produces an even starker contrast, with 88% in Scotland and 65% in England providing a positive evaluation of local authorities. A full range of actors was viewed more positively in Scotland than in England: civil society organisations (82% against 57%), the Syrian community (89% against 69%) and other migrant populations (78% against 59%). What is most striking in these results though is the evaluation of the general population, with 61% of Syrians in England rating the overall population positively, compared to 90% in Scotland who did similarly. This could be suggestive of differing views between Scotland and England about Syrian refugees (though we will be testing this in future work). What we can say now is that we suspect that the less anti-immigration narrative in Scotland, especially in terms of political leadership and the media, does appear to have an impact on how refugees perceive the general population.

Different experiences of settlement and social citizenship also impact refugees’ general happiness and, indeed, desire to settle in the UK in the longer term. Young Syrians in England are more confident than those in Scotland (32% compared to 19%), perhaps, a by-product of the different ways in which the policy of self-reliance manifests itself in the two nations. However, young Syrians appear to be much happier in Scotland than in England (49% against 30%). These emotions feed directly into young refugees’ plans for their future in the UK, with 81% of those in Scotland intending to remain in the country, compared to 65% of those in England.

Experiences of social citizenship in the form of welfare regimes should therefore not be viewed narrowly or characterised simply as a ‘nicer’ thing to do or a more ‘migrant-friendly’ thing to do. Territorial variance in how welfare stratification is experienced has far-reaching implications for refugees’ outlook on life and long-term planning.

This article contributes to an emerging literature on devolution and migration (see Hepburn and Zapata-Barrero, 2014), and indeed to debates about both the uploading and downloading of immigration controls to both public and private bodies in the form of internal and everyday bordering. What we have shown is that even in a state where immigration control is highly centralised, spaces of territorial differentiation are possible. Clearly as far as the experiences of refugees are concerned whether this variance is positive or negative depends on the politics of the various levels of Government. What this might suggest is that states with federal systems or more entrenched forms of devolution, and indeed those where immigrant policy is more explicitly devolved are a potentially fruitful comparison.

## Supplemental Material

Supplemental Material - Territorial variance in the UK’s refugee politics and its consequences: Young Syrian refugees in England and Scotland Supplemental Material for Territorial variance in the UK’s refugee politics and its consequences: Young Syrian refugees in England and Scotland by Gareth Mulvey, Dimitris Skleparis and Brian Boyle in Environment and Planning C: Politics and Space

## Note

1 In the absence of a universally accepted definition of ’youth’, and to obviate the need for parental consent, we have adapted the UN Habitat’s (2011) 15–32 age range to focus on older youth from 18 to 32.
