# Supplementary material for: Territorial variance in the UK’s refugee politics and its consequences: Young Syrian refugees in England and Scotland
Source: Environ Plan C Polit Space. 2023 Mar 29;41(5):958–75. doi: 10.1177/23996544231165440 (PMC13086280; doi:10.1177/23996544231165440)
Supplement: Supplemental Material - Territorial variance in the UK’s refugee politics and its consequences: Young Syrian refugees in England and Scotland [file sj-pdf-1-epc-10.1177_23996544231165440.pdf]

Appendix

List of tables

1.0 Welfare Provisions and Country of Settlement

- Table 1.1 Accommodation funding by country of settlement
- Table 1.2 Accommodation type by country of settlement
- Table 1.3 Main source of income (Top choice) by country of settlement
- Table 1.4 Benefited from English language course provision by country of settlement

2.0 Welfare Provisions and Route of Access

- Table 2.1 Accommodation funding by route of access
- Table 2.2 Type of accommodation by route of access
- Table 2.3 Main source of income (First choice) by route of access
- Table 2.4 Benefited from English language course provision by route of access

3.0 Employment and Country of Settlement

- Table 3.1 Employment status by country of settlement
- Table 3.2 Employment status by country of settlement (Employment dummy variable)
- Table 3.3 Access to social security by country of settlement (of those in employment)
- Table 3.4 Employed in professional/highly skilled work by country of settlement
- Table 3.5 Employed in skilled manual work by country of settlement

4.0 Employment and Gender

- Table 4.1 Employment status by gender
- Table 4.2 Employment status by gender (Employment dummy variable)

5.0 Employment and Route of Access

- Table 5.1 Employment status by route of access
- Table 5.2 Employment status (dummy) by country of settlement and route of access

6.0 Education

- Table 6.1 Evidence of qualifications by country of settlement
- Table 6.2 University education by country of settlement
- Table 6.4 Current student status by country of settlement
- Table 6.5 Current student status by route of access
- Table 6.6 Current student status by gender

7.0 Age, Children, and Aspirations

- Table 7 Evidence of qualifications by country of settlement

7.0 Past, Aspired, and Current Employment/Student Status by Country of Settlement
- Table 7.1 Aspired student status by country of settlement
- Table 7.2 Past student status by country of settlement
- Table 7.3 Aspired employment status by country of settlement
- Table 7.4 Past employment status by country of settlement
- Table 7.5 Past versus current employment rates of young Syrian refugees (full sample)
- Table 7.6 Current employment by country of settlement (for those who were previously in employment only)
- Table 7.8 Current employment by country of settlement (for those who aspire to be in employment only)
- Table 7.7 Aspired versus current employment rates of young Syrian refugees (full sample)

8.0 Past, Aspired, and Current Highly Skilled Work
- Table 8.1 Highly skilled/professional work aspirations by country of settlement
- Table 8.3 Highly skilled/professional previous work experience by country of settlement

9.0 Past, Aspired, and Current Employment/Student Status by Route of Access
- Table 9.1 Aspired employment status by country of settlement and route of access
- Table 9.2 Aspired student status by country of settlement and route of access

10.0 Evaluations
- Table 10.1 Evaluation of the national government by country of settlement
- Table 10.2 Evaluation of the local authority by country of settlement
- Table 10.3 Evaluation of civil society organisations by country of settlement
- Table 10.4 Evaluation of the British public by country of settlement
- Table 10.5 Evaluation of the Syrian community by country of settlement
- Table 10.6 Evaluation of other migrant population by country of settlement

11.0 Emotions
- Table 11.1 Feelings about current situation: Confidence, by country of settlement
- Table 11.2 Feelings about current situation: Happiness, by country of settlement

12.0 Remaining in the UK
- Table 12. Plans on remaining in the UK by country of settlement

13.0 Age, having a child in the UK, and aspirations
- Figure 1. Respondent age by country of settlement
- Figure 2. Employment and educational aspirations by age
- Table 13.1. Has child in the UK by country of settlement
- Table 13.2. Has child in the UK by employment aspirations
- Table 13.3. Has child in the UK by educational aspirations

Table 1.1 Accommodation funding by country of settlement

Country of settlement	*Who pays for your accommodation?*			*Total*
	I pay rent	State funded	Other	
England	72<br>34.8 %	89<br>43 %	46<br>22.2 %	207<br>100 %
Scotland	45<br>17.2 %	181<br>69.3 %	35<br>13.4 %	261<br>100 %
Total	117<br>25 %	270<br>57.7 %	81<br>17.3 %	468<br>100 %

$\chi^2 = 33.285 \cdot df = 2 \cdot p < 0.001$

Table 1.2 Accommodation type by country of settlement

Country of settlement	*Accommodation type*		*Total*
	Shared	Not Shared	
England	58<br>29.9 %	136<br>70.1 %	194<br>100 %
Scotland	29<br>11.5 %	224<br>88.5 %	253<br>100 %
Total	87<br>19.5 %	360<br>80.5 %	447<br>100 %

$\chi^2 = 25.287 \cdot df = 1 \cdot p < 0.001$

Table 1.3 Main source of income (Top choice) by country of settlement

Country of settlement	*Main source of income*		*Total*
	Government welfare	Other	
England	74<br>34.9 %	138<br>65.1 %	212<br>100 %
Scotland	173<br>65.3 %	92<br>34.7 %	265<br>100 %
Total	247<br>51.8 %	230<br>48.2 %	477<br>100 %

$\chi^2 = 43.529 \cdot df=1 \cdot p < 0.001$

Table 1.4 Benefited from English language course provision by country of settlement

Country of settlement	***Benefited from English language course provision***		***Total***
	Yes	No	
England	115<br>54.2 %	97<br>45.8 %	212<br>100 %
Scotland	195<br>73.6 %	70<br>26.4 %	265<br>100 %
Total	310<br>65 %	167<br>35 %	477<br>100 %

$\chi^2 = 19.361 \cdot df=1 \cdot p < 0.001$

Route of access, Country of Settlement, and Welfare Provisions

Log linear analysis was used to explore the association between all three categorical variables of interest. None of the three-way interactions with route of access, country of settlement, and any of the welfare variables were statistically significant.

Who pays for Accommodation
The likelihood ratio statistic for the final model was χ^2 (1) = 0.4, p = .51. The highest order interaction between all three variables, as well as the interaction between route of access and country of settlement was not statistically significant. The remaining second order interactions were statistically significant (Country of settlement * Accommodation type) χ^2 (1) = 28.9, p < .001, (Route of access * Accommodation type) χ^2 (1) = 10.2, p = .001.

Shared Accommodation
The likelihood ratio statistic for the final model was χ^2 (1) = 0.4, p = .51. The highest order interaction between all three variables, as well as the interaction between route of access and country of settlement was not statistically significant. The remaining second order interactions were statistically significant (Country of settlement * Accommodation type) χ^2 (1) = 28.9, p < .001, (Route of access * Accommodation type) χ^2 (1) = 10.2, p = .001.

Government Welfare Payments
The likelihood ratio statistic for the final model was χ^2 (1) = 1.1, p = .29. The highest order interaction between all three variables, as well as the interaction between route of access and country of settlement was not statistically significant. The remaining second order interactions were statistically significant (Country of settlement * Welfare payments) χ^2 (1) = 28.3, p < .001, (Route of access * Welfare payments) χ^2 (1) = 56.2, p < .001.

English language Course Provision
The likelihood ratio statistic for the final model was χ^2 (1) = 0.25, p = .62. The highest order interaction between all three variables was not statistically significant. The remaining second order interactions were statistically significant (Country of settlement * ELC Provision) χ^2 (1) = 6.7, p < .001, (Route of access * ELC Provision) χ^2 (1) = 19.6, p < .001, (Route of access * Country of settlement) χ^2 (1) = 5.6, p < .05.

The chi-square tables below display the relationship between route of access and each of the welfare variables.

Table 2.1 Accommodation funding by route of access

Route of access	*Who pays for your accommodation?*			*Total*
	I pay rent	State funded	Other	
Asylum route	76<br>38.8 %	94<br>48 %	26<br>13.3 %	196<br>100 %
Resettlement	20<br>10.9 %	148<br>80.9 %	15<br>8.2 %	183<br>100 %
Total	96<br>25.3 %	242<br>63.9 %	41<br>10.8 %	379<br>100 %

$\chi^2=47.277 \cdot df=2 \cdot p < 0.001$

Table 2.2 Type of accommodation by route of access

Route to refugee status	*Accommodation type*		*Total*
	Shared	Not Shared	
Asylum route	51<br>26.6 %	141<br>73.4 %	192<br>100 %
Resettlement	20<br>10.9 %	163<br>89.1 %	183<br>100 %
Total	71<br>18.9 %	304<br>81.1 %	375<br>100 %

$\chi^2=14.919 \cdot df=1 \cdot p < 0.001$

Table 2.3 Main source of income (First choice) by route of access

Route of access	*Main source of income*		*Total*
	Government welfare	Other	
Asylum route	73<br>36.9 %	125<br>63.1 %	198<br>100 %
Resettlement	144<br>77 %	43<br>23 %	187<br>100 %

Total	217<br>56.4 %	168<br>43.6 %	385<br>100 %

χ^2= 62.991· df=1 · p < 0.001

Table 2.4 Benefited from English language course provision by route of access

Route of access	***Benefited from English language course provision***		***Total***
	Yes	No	
Asylum route	122<br>61.6 %	76<br>38.4 %	198<br>100 %
Resettlement	156<br>83.4 %	31<br>16.6 %	187<br>100 %
Total	278<br>72.2 %	107<br>27.8 %	385<br>100 %

χ^2= 22.788 · df=1 · p < 0.001

Country of	*Employment status*	*Total*

Table 3.1 Employment status by country of settlement

Table 3.2 Employment status by country of settlement (Employment dummy variable)

Country of settlement	***Currently employed***		***Total***
	Yes	No	
England	71<br>33.6 %	140<br>66.4 %	211<br>100 %
Scotland	53<br>20.3 %	208<br>79.7 %	261<br>100 %
Total	124<br>26.3 %	348<br>73.7 %	472<br>100 %

$\chi^2=10.724 \cdot df=1 \cdot p=0.001$

	Employed	Unable to work/ Unemployed, not looking for a job	Student	Homemaker	Unemployed, looking for job	
England	71<br>33.6 %	19<br>9 %	68<br>32.2 %	22<br>10.4 %	31<br>14.7 %	211<br>100 %
Scotland	53<br>20.3 %	12<br>4.6 %	94<br>36 %	44<br>16.9 %	58<br>22.2 %	261<br>100 %
Total	124<br>26.3 %	31<br>6.6 %	162<br>34.3 %	66<br>14 %	89<br>18.9 %	472<br>100 %

χ^2=18.805 · df=4 · p < 0.001

Country of settlement	***Do you have social security?***		***Total***
	Yes	No	
England	28<br>47.5 %	31<br>52.5 %	59<br>100 %
Scotland	28<br>70 %	12<br>30 %	40<br>100 %
Total	56<br>56.6 %	43<br>43.4 %	99<br>100 %

χ^2=4.93 · df=1 · p < 0.05

Table 3.3 Access to social security by country of settlement (of those in employment)

Table 3.4 Employed in professional/highly skilled work by country of settlement

Country of settlement	***Employed in professional/highly skilled work***		***Total***
	Yes	No	
England	16<br>22.9 %	54<br>77.1 %	70<br>100 %
Scotland	13<br>24.5 %	40<br>75.5 %	53<br>100 %

Total	29<br>23.6 %	94<br>76.4 %	123<br>100 %

χ^2=0.047 · df=1 · p = 0.829

Table 3.5 Employed in skilled manual work by country of settlement

Country of settlement	***Employed in skilled manual work***		***Total***
	Yes	No	
England	12<br>17.1 %	58<br>82.9 %	70<br>100 %
Scotland	15<br>28.3 %	38<br>71.7 %	53<br>100 %
Total	27<br>22 %	96<br>78 %	123<br>100 %

χ^2= 2.192· df=1 · p = 0.139

Table 4.1 Employment status by gender

	Employment status					
Gender	Employed	Unable to work/ Unemployed, not looking for a job	Student	Homemaker	Unemployed, looking for a job	***Total***
Male	97<br>32.4 %	19<br>6.4 %	106<br>35.5 %	0<br>0 %	77<br>25.8 %	299<br>100 %
Female	27<br>16.1 %	8<br>4.8 %	56<br>33.3 %	66<br>39.3 %	11<br>6.5 %	168<br>100 %
Total	124<br>26.6 %	27<br>5.8 %	162<br>34.7 %	66<br>14.1 %	88<br>18.8 %	467<br>100 %

χ^2=149.984 · df=4 · p < 0.001

Gender, Country of settlement, and Employment Status

A log linear analysis was conducted to explore the association between all three variables of interest. The likelihood ratio statistic for the final model was χ^2 (2) = 3.52, p = .172. The highest order interaction between all three variables, as well as the interaction between gender and country of settlement were not statistically significant. The remaining second order interactions were statistically significant (Country of settlement * Employment status) χ^2 (1) = 13.5, p < .001, (Gender * Employment status) χ^2 (1) = 17.3, p < .001.

The lack of any significant effect for the three-way interaction term implies that relationship between gender and employment status did not differ significantly depending on which country individuals settled in.

Table 4.2 Employment status by gender (Employment dummy variable)

Gender	*Employment status*		***Total***
	Yes	No	
Male	97<br>32.4 %	202<br>67.6 %	299<br>100 %
Female	27<br>16.1 %	141<br>83.9 %	168<br>100 %
Total	124<br>26.6 %	343<br>73.4 %	467<br>100 %

χ^2=14.78 · df=1 · p = 0.001

Table 5.1 Employment status by route of access

Route of access	***Employment status***					***Total***
	Employed	Unable to work/ Unemployed, not looking for a job	Student	Homemaker	Unemployed, looking for a job	
Asylum route	82<br>41.6 %	9<br>4.6 %	60<br>30.5 %	13<br>6.6 %	33<br>16.8 %	197<br>100 %
Resettlement	17<br>9.1 %	9<br>4.8 %	72<br>38.7 %	38<br>20.4 %	50<br>26.9 %	186<br>100 %
Total	99<br>25.8 %	18<br>4.7 %	13<br>2<br>34.5 %	51<br>13.3 %	83<br>21.7 %	383<br>100 %

χ^2=59.237 · df=4 · p < 0.001

Table 5.2 Employment status (dummy) by country of settlement and route of access

Country of settlement	Route to refugee status	Currently employed: Yes	No	Total
England	Asylum route	46<br>47.9 %<br>79.3 %	50<br>52.1 %<br>50 %	96<br>100 %<br>60.8 %
	Resettlement	12<br>19.4 %<br>20.7 %	50<br>80.6 %<br>50 %	62<br>100 %<br>39.2 %
	Total	58<br>36.7 %<br>100 %	100<br>63.3 %<br>100 %	158<br>100 %<br>100 %
Scotland	Asylum route	36<br>35.6 %<br>87.8 %	65<br>64.4 %<br>35.3 %	101<br>100 %<br>44.9 %
	Resettlement	5<br>4 %<br>12.2 %	119<br>96 %<br>64.7 %	124<br>100 %<br>55.1 %

Total	41<br>18.2 %<br>100 %	184<br>81.8 %<br>100 %	225<br>100 %<br>100 %

n = 383

Chi-sq - England
χ^2=13.227 · df=1 · p=0.001
OR = 3.8 (1.7, 8.9) 95% CI

Chi-sq – Scotland
χ^2=37.326 · df=1 · p < 0.001
OR = 13 (4.8, 44.6) 95% CI

A log linear analysis was conducted to explore the association between all three variables of interest. The final model contained all effects and had a likelihood ratio statistic of χ^2 (0) = 0, p = 1. The highest order interaction between all three variables (country of settlement, route to refugee status, and employment status) was statistically significant χ^2 (1) = 4.1, p = .043.

Separate chi-square tests were run for both those settled in England, and those in Scotland, to examine these effects further. For those settled in England (χ^2(1) = 13.2, p < .001), there was a significant relationship between the route to refugee status and employment, as was the case for those settled in Scotland (χ^2(1) = 37.3, p < .001). This implies that young refugees that gained refugee status via the asylum route were more likely to be in employment than those who entered via resettlement, especially for those who settled in Scotland.

Table 6.1 Evidence of qualifications by country of settlement

Country of settlement	***Documentation of qualifications***<br>Yes	 No	***Total***
England	128<br>63.1 %	75<br>36.9 %	203<br>100 %
Scotland	104<br>40.3 %	154<br>59.7 %	258<br>100 %
Total	232<br>50.3 %	229<br>49.7 %	461<br>100 %

χ^2= 23.509 · df=1 · p < 0.001

Table 6.2 University education by country of settlement

Country of settlement	*University educated*<br>Yes	 No	***Total***

England	69<br>37.3 %	116<br>62.7 %	185<br>100 %
Scotland	51<br>20 %	204<br>80 %	255<br>100 %
Total	120<br>27.3 %	320<br>72.7 %	440<br>100 %

χ^2=16.173· df=1 · p < 0.001

Table 6.4 Current student status by country of settlement

Country of settlement	***Currently a student***		***Total***
	Yes	No	
England	68<br>32.2 %	143<br>67.8 %	211<br>100 %
Scotland	94<br>36 %	167<br>64 %	261<br>100 %
Total	162<br>34.3 %	310<br>65.7 %	472<br>100 %

χ^2= 0.743· df=1 · p=0.389

Table 6.5 Current student status by route of access

Route of access	***Currently a student***		***Total***
	Yes	No	

Asylum route	60<br>30.5 %	137<br>69.5 %	197<br>100 %
Resettlement	72<br>38.7 %	114<br>61.3 %	186<br>100 %
Total	132<br>34.5 %	251<br>65.5 %	383<br>100 %

χ^2= 2.885 · df=1 ·p=0.112

Table 6.6 Current student status by gender

Gender	***Currently a student***		***Total***
	Yes	No	
Male	106<br>35.5 %	193<br>64.5 %	299<br>100 %
Female	56<br>33.3 %	112<br>66.7 %	168<br>100 %
Total	162<br>34.7 %	305<br>65.3 %	467<br>100 %

χ^2= 0.213 · df=1 · p = 0.614

Table 7.1 Aspired student status by country of settlement

Country of settlement	***Aspires to student status***		***Total***
	Yes	No	
England	58<br>31.5 %	126<br>68.5 %	184<br>100 %
Scotland	79<br>33.2 %	159<br>66.8 %	238<br>100 %
Total	137<br>32.5 %	285<br>67.5 %	422<br>100 %

χ^2= 0.132 · df=1 · p=0.716

Table 7.2 Past student status by country of settlement

Country of settlement	***Was a student 6-months prior to leaving Syria***		***Total***
	Yes	No	
England	73<br>36.9 %	125<br>63.1 %	198<br>100 %
Scotland	102<br>39.7 %	155<br>60.3 %	257<br>100 %
Total	175<br>38.5 %	280<br>61.5 %	455<br>100 %

χ^2= 0.376· df=1 · p=0.54

Table 7.3 Aspired employment status by country of settlement

Country of settlement	***Aspires to be in Employment***		***Total***
	Yes	No	
England	114<br>62 %	70<br>38 %	184<br>100 %
Scotland	140<br>58.8 %	98<br>41.2 %	238<br>100 %
Total	254<br>60.2 %	168<br>39.8 %	422<br>100 %

χ^2= 0.425 · df=1 · p=0.514

Table 7.4 Past employment status by country of settlement

Country of settlement	*Employed 6-months prior to leaving Syria*		*Total*
	Yes	No	
England	99<br>50 %	99<br>50 %	198<br>100 %
Scotland	103<br>40.1 %	154<br>59.9 %	257<br>100 %
Total	202<br>44.4 %	253<br>55.6 %	455<br>100 %

χ^2= 4.460· df=1 · $p < 0.05$

7.5 Past versus current employment rates of young Syrian refugees (full sample)

Previously employed	*Currently employed*		***Total***
	Yes	No	
Yes	82<br>41 %	118<br>59 %	200<br>100 %
No	40<br>16 %	210<br>84 %	250<br>100 %
Total	122<br>27.1 %	328<br>72.9 %	450<br>100 %

χ^2=35.142 · df=1 $p < 0.001$

7.6 Current employment by country of settlement (for those who were previously in employment only)

Country of settlement	***Currently employed***		***Total***
	Yes	No	
England	47<br>48 %	51<br>52 %	98<br>100 %
Scotland	35<br>34.3 %	67<br>65.7 %	102<br>100 %
Total	82<br>41 %	118<br>59 %	200<br>100 %

$\chi^2 = 3.847 \cdot df=1 \cdot p < 0.05$

7.7 Aspired versus current employment rates of young Syrian refugees (full sample)

Aspires to be employed	***Currently employed***		***Total***
	Yes	No	
Yes	98<br>39.2 %	152<br>60.8 %	250<br>100 %
No	10<br>6 %	157<br>94 %	167<br>100 %
Total	108<br>25.9 %	309<br>74.1 %	417<br>100 %

$\chi^2=57.544 \cdot df=1 \cdot p < 0.001$

7.8 Current employment by country of settlement (for those who aspire to be in employment only)

Country of settlement	*Currently employed*		*Total*
	Yes	No	
England	61<br>54 %	52<br>46 %	113<br>100 %
Scotland	37<br>27 %	100<br>73 %	137<br>100 %
Total	98<br>39.2 %	152<br>60.8 %	250<br>100 %

χ^2=18.906 · df=1 · p < 0.001

Table 8.1 Highly skilled/professional work aspirations by country of settlement

Country of settlement	*Aspires to be employed in highly skilled/professional job*		*Total*
	Yes	No	
England	48<br>42.9 %	64<br>57.1 %	112<br>100 %
Scotland	29<br>20.7 %	111<br>79.3 %	140<br>100 %
Total	77<br>30.6 %	175<br>69.4 %	252<br>100 %

χ^2=14.378 · df=1 · p < 0.001

Table 8.3 Highly skilled/professional previous work experience by country of settlement

Country of settlement	***Previously employed in highly skilled/professional job***		***Total***
	Yes	No	
England	43<br>44.3 %	54<br>55.7 %	97<br>100 %
Scotland	22<br>21.4 %	81<br>78.6 %	103<br>100 %
Total	65<br>32.5 %	135<br>67.5 %	200<br>100 %

χ^2=12.015 · df=1 · p < 0.001

Table 9.1 Aspired employment status by country of settlement and route of access

Country of settlement	***Route of access***	***Aspires to be in employment***		***Total***
		Yes	No	
England	Asylum route	65<br>73.9 %<br>74.7 %	23<br>26.1 %<br>41.8 %	88<br>100 %<br>62 %
	Resettlement	22<br>40.7 %<br>25.3 %	32<br>59.3 %<br>58.2 %	54<br>100 %<br>38 %
	Total	87<br>61.3 %<br>100 %	55<br>38.7 %<br>100 %	142<br>100 %<br>100 %

Scotland	Asylum route	54<br>59.3 %<br>42.9 %	37<br>40.7 %<br>45.1 %	91<br>100 %<br>43.8 %
	Resettlement	72<br>61.5 %<br>57.1 %	45<br>38.5 %<br>54.9 %	117<br>100 %<br>56.2 %
	Total	126<br>60.6 %<br>100 %	82<br>39.4 %<br>100 %	208<br>100 %<br>100 %

Chi-sq - England
$\chi^2 = 15.472 \cdot df=1 \cdot p < 0.001$

Chi-sq – Scotland
$\chi^2 = 0.104 \cdot df=1 \cdot p = 0.748$

Likelihood ratio statistic for final model that contained all effects χ^2 (0) = 0 , p = 1. This indicates that the three-way interaction between aspired employment, country of settlement, and route of access was statistically significant (χ^2 (1) = 10.65, p = .001).

The Chi-square tests indicate that in England, those entering via the asylum route were significantly more likely to aspire to be in employment, compared to those via resettlement. Meanwhile, for those settled in Scotland, the differences between the two groups were not statistically significant.

Table 9.2 Aspired student status by country of settlement and route of access

Country of settlement	***Route of access***	***Aspires to be a student***<br>Yes	 No	***Total***
England	Asylum route	18<br>20.5 %<br>39.1 %	70<br>79.5 %<br>72.9 %	88<br>100 %<br>62 %
	Resettlement	28<br>51.9 %<br>60.9 %	26<br>48.1 %<br>27.1 %	54<br>100 %<br>38 %
	Total	46<br>32.4 %<br>100 %	96<br>67.6 %<br>100 %	142<br>100 %<br>100 %

Scotland		34	57	91
	Asylum route	37.4 %	62.6 %	100 %
		52.3 %	39.9 %	43.8 %
		31	86	117
	Resettlement	26.5 %	73.5 %	100 %
		47.7 %	60.1 %	56.2 %
		65	143	208
	Total	31.2 %	68.8 %	100 %
		100 %	100 %	100 %

Chi-sq - England
χ^2=15.063 · df=1 · p < 0.001

Chi-sq – Scotland
χ^2= 2.814· df=1 · p = 0.09

Likelihood ratio statistic for final model that contained all effects χ^2 (0) = 0 , p = 1. This indicates that the three-way interaction between aspired student status, country of settlement, and route of access was statistically significant (χ^2 (1) = 16.38, p < .001).

The Chi-square tests indicate that in England, those entering via the asylum route were significantly less likely to aspire to be a student, compared to those via resettlement. Meanwhile, for those settled in Scotland, it was the asylum group who were more likely to aspire to student status, however, the difference between the two groups was not statistically significant.

Country of Settlement and Route of Access – Log-linear Models

Aspired Employment Status
Likelihood ratio statistic for final model that contained all effects χ^2 (0) = 0 , p = 1. This indicates that the three-way interaction between aspired employment, country of settlement, and route of access was statistically significant (χ^2 (1) = 10.65, p = .001). The Chi-square tests indicate that in England, those entering via the asylum route were significantly more likely to aspire to be in employment, compared to those via resettlement. Meanwhile, for those settled in Scotland, the differences between the two groups were not statistically significant.

Current Employment Status
The likelihood ratio statistic for the final model was χ^2 (0) = 0, p = 1. The highest order interaction between all three variables was statistically significant χ^2 (1) = 4.1, p < .05. This indicates that in Scotland, those from the asylum route are significantly more likely to be in employment compared to those from resettlement. While this is also the case in England, the strength of the relationship between route of access and employment is significantly weaker.

Past Employment Status
The likelihood ratio statistic for the final model was χ^2 (1) = 2.1, p = 0.147. The highest order interaction between all three variables was not statistically significant, nor was the interaction between country of settlement and previous employment. The remaining second order interactions were statistically significant (Country of settlement * Route of access) χ^2 (1) = 9.8, p = .001, (Route of access * Previous employment) χ^2 (1) = 7.15, $p < .01$. This implies that while those entering via the Asylum route were more likely to have previously been in employment compared to those from the resettlement route, this relationship did not differ significantly depending on the country of settlement.

Aspired Student Status
Likelihood ratio statistic for final model that contained all effects χ^2 (0) = 0 , p = 1. This indicates that the three-way interaction between aspired student status, country of settlement, and route of access was statistically significant (χ^2 (1) = 16.38, $p < .001$). The Chi-square tests indicate that In England, those entering via the asylum route were significant less likely to aspire to be a student, compared to those via resettlement. Meanwhile, for those settled in Scotland, it was the asylum group who were more likely to aspire to student status, however, the difference between the two groups was not statistically significant.

Current Student Status
The likelihood ratio statistic for the final model was χ^2 (1) = 2.1, p = 0.147. The highest order interaction between all three variables was not statistically significant, nor was the interaction between country of settlement and student status, or route of access and student status.
The only statistically significant interaction was Country of settlement * Route of access, χ^2 (1) = 8.88, $p < .01$. This indicates that neither country or settlement, nor route of access is significantly related to young refugees' current student status.

Past Student Status
The likelihood ratio statistic for the final model was χ^2 (0) = 0, p = 1. The highest order interaction between all three variables was statistically significant χ^2 (1) = 6.3, $p < .05$.
The difference in previous student rates for those settled in England did not differ significantly between those from either the resettlement or asylum route. In Scotland, however, those entering via the asylum route were significantly more likely to have previously been a student, compared to those from the resettlement route.

Table 10.1 Evaluation of the national government by country of settlement

Country of settlement	*Evaluation of the national government*			*Total*
	Positive	Neutral	Negative	
England	121<br>69.9 %	40<br>23.1 %	12<br>6.9 %	173<br>100 %
Scotland	202<br>89 %	21<br>9.3 %	4<br>1.8 %	227<br>100 %
Total	323<br>80.8 %	61<br>15.2 %	16<br>4 %	400<br>100 %

$\chi^2 = 23.367 \cdot df=2 \cdot p < 0.001$

Table 10.2 Evaluation of the local authority by country of settlement

Country of settlement	*Evaluation of the local authority*			*Total*
	Positive	Neutral	Negative	
England	109<br>65.3 %	45<br>26.9 %	13<br>7.8 %	167<br>100 %
Scotland	210<br>87.9 %	22<br>9.2 %	7<br>2.9 %	239<br>100 %
Total	319<br>78.6 %	67<br>16.5 %	20<br>4.9 %	406<br>100 %

$\chi^2 = 29.844 \cdot df=2 \cdot p < 0.001$

Table 10.3 Evaluation of civil society organisations by country of settlement

Country of settlement	*Evaluation of civil society organisations*			*Total*
	Positive	Neutral	Negative	
England	90<br>57.3 %	58<br>36.9 %	9<br>5.7 %	157<br>100 %
Scotland	149<br>82.3 %	25<br>13.8 %	7<br>3.9 %	181<br>100 %

Total	239<br>70.7 %	83<br>24.6 %	16<br>4.7 %	338<br>100 %

χ^2= 26.364 · df=2 · p < 0.001

Table 10.4 Evaluation of the British public by country of settlement

Country of settlement	***Evaluation of the British public***			***Total***
	Positive	Neutral	Negative	
England	110<br>60.8 %	60<br>33.1 %	11<br>6.1 %	181<br>100 %
Scotland	228<br>90.1 %	22<br>8.7 %	3<br>1.2 %	253<br>100 %
Total	338<br>77.9 %	82<br>18.9 %	14<br>3.2 %	434<br>100 %

χ^2= 52.888 · df=2 · p < 0.001

Table 10.5 Evaluation of the Syrian community by country of settlement

Country of settlement	***Evaluation of the Syrian community***			***Total***
	Positive	Neutral	Negative	
England	122<br>68.5 %	42<br>23.6 %	14<br>7.9 %	178<br>100 %
Scotland	223<br>88.8 %	15<br>6 %	13<br>5.2 %	251<br>100 %
Total	345<br>80.4 %	57<br>13.3 %	27<br>6.3 %	429<br>100 %

χ^2= 30.867· df=2 · p < 0.001

Table 10.6 Evaluation of other migrant population by country of settlement

Country of settlement	***Evaluation of other migrant populations***			***Total***
	Positive	Neutral	Negative	
England	99<br>58.9 %	64<br>38.1 %	5<br>3 %	168<br>100 %
Scotland	166<br>77.6 %	40<br>18.7 %	8<br>3.7 %	214<br>100 %

Total	265<br>69.4 %	104<br>27.2 %	13<br>3.4 %	382<br>100 %

χ^2= 17.891· df=2 · p < 0.001

Table 11.1 Feelings about current situation: Confidence, by country of settlement

Country of settlement	***Which comes closest to describing your feelings about your current situation in the UK: 'Confident'***		***Total***
	Yes	No	
England	68<br>32.1 %	144<br>67.9 %	212<br>100 %
Scotland	50<br>18.9 %	215<br>81.1 %	265<br>100 %
Total	118<br>24.7 %	359<br>75.3 %	477<br>100 %

χ^2= 11.035 · df=1 · p < 0.001

Table 11.2 Feelings about current situation: Happiness, by country of settlement

Country of settlement	***Which comes closest to describing your feelings about your current situation in the UK: 'Happy'***		***Total***
	Yes	No	
England	63<br>29.7 %	149<br>70.3 %	212<br>100 %
Scotland	131<br>49.4 %	134<br>50.6 %	265<br>100 %
Total	194<br>40.7 %	283<br>59.3 %	477<br>100 %

χ^2= 18.975 · df=1 · p < 0.001

Table 12. Plans on remaining in the UK by country of settlement

Country of settlement	***Do you plan on remaining in the UK?***			***Total***
	Yes	No	Don't know	
England	117<br>65.4 %	35<br>19.6 %	27<br>15.1 %	179<br>100 %
Scotland	207<br>80.5 %	21<br>8.2 %	29<br>11.3 %	257<br>100 %
Total	324<br>74.3 %	56<br>12.8 %	56<br>12.8 %	436<br>100 %

$\chi^2=15.1 \cdot df=2 \cdot p < 0.001$

13.0 Age, having a child in the UK, and aspirations

There was a consistent pattern that older respondents, as well as those with a child were more likely to aspire to be in employment, while the opposite pattern was true for educational aspirations (younger and childless respondents were significantly more likely to aspire to be students).

There were no statistically significant differences between respondents settled in Scotland or England in terms of whether they have a child with them in the UK (46%, 41%), or age.

Figure 1 Respondent Age by Country of Settlement

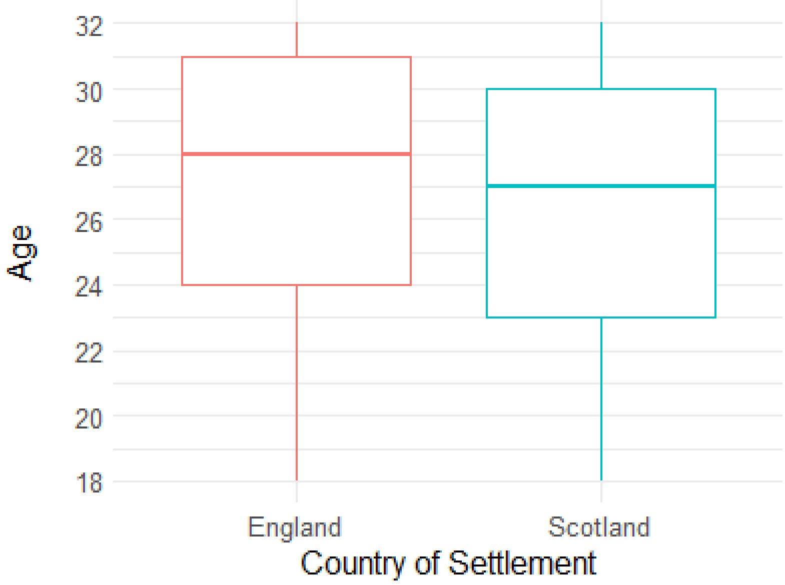

2-sided t-test between age and country of settlement was not statistically significant (p = 0.076).

Figure 2 Aspirations to be in Employment and Education by Age

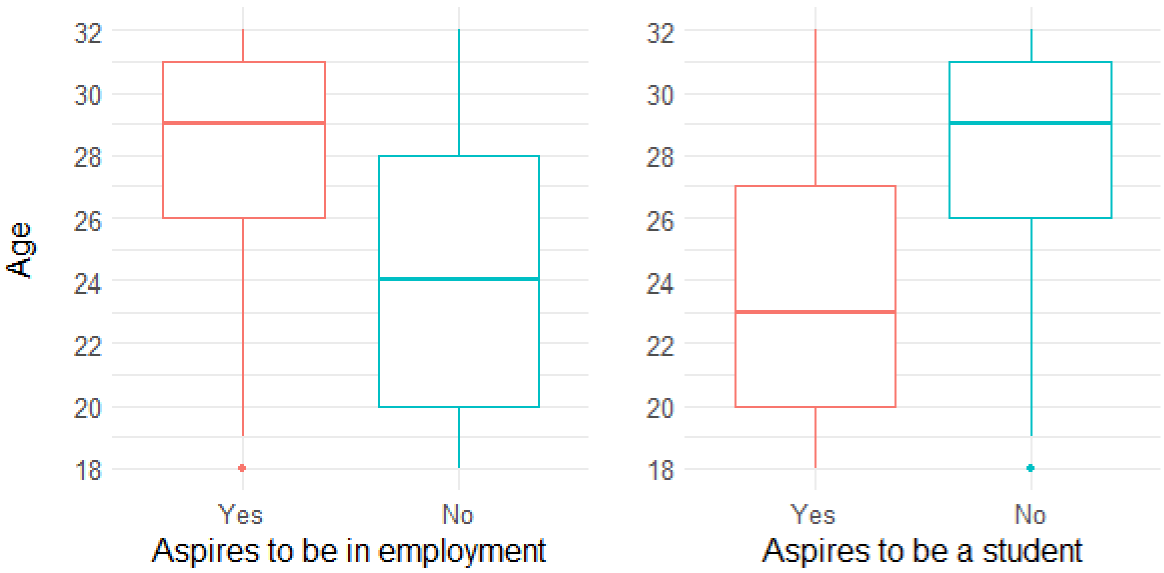

2-sided t-tests between age and employment aspirations, and age and education aspirations were both statistically significant at the $p < .001$ level.

13.1 Has child in the UK by country of settlement

Country of settlement	*Has a child with them in the UK*		***Total***
	No	Yes	
England	123<br>59.4 %	84<br>40.6 %	207<br>100 %
Scotland	142<br>54 %	121<br>46 %	263<br>100 %
Total	265<br>56.4 %	205<br>43.6 %	470<br>100 %

$\chi^2=1.176 \cdot df=1 \cdot \varphi=0.054 \cdot p=0.278$

13.2 Has child in the UK by employment aspirations

Has a child with them in the UK	*Aspires to be in Employment*		***Total***
	Yes	No	
No	121<br>51.5 %	114<br>48.5 %	235<br>100 %
Yes	132<br>71.4 %	53<br>28.6 %	185<br>100 %
Total	253<br>60.2 %	167<br>39.8 %	420<br>100 %

χ^2=16.230 · df=1 · φ=0.201 · p<0.001

13.3 Has child in the UK by education aspirations

Has a child with them in the UK	*Aspires to be a Student*		***Total***
	Yes	No	
No	111<br>47.2 %	124<br>52.8 %	235<br>100 %
Yes	25<br>13.5 %	160<br>86.5 %	185<br>100 %
Total	136<br>32.4 %	284<br>67.6 %	420<br>100 %

χ^2=52.226 · df=1 · φ=0.358 · p<0.001

Rerunning the tests using a combined variable for whether a respondent has a child with them in the UK, **or** back in Syria, **or** in another country yields similar results to the UK only variable displayed above.
